# Asymptomatic low pulse oximetry measurements in leprosy patients in the time of COVID-19: Dapsone side effect

**DOI:** 10.1590/0037-8682-0491-2021

**Published:** 2022-02-25

**Authors:** Marco Andrey Cipriani Frade, Fred Bernardes, Ana Laura Quirino de Lima, Marcelo Bezerra de Menezes, Helena Barbosa Lugão

**Affiliations:** 1 Universidade de São Paulo, Faculdade de Medicina de Ribeirão Preto, Divisão de Dermatologia, Departamento de Clínica Médica, Ribeirão Preto, SP, Brasil.; 2 Universidade de São Paulo, Faculdade de Medicina de Ribeirão Preto, Hospital das Clínicas, Centro de Referência Nacional em Dermatologia Sanitária com Ênfase em Hanseníase, Ribeirão Preto, SP, Brasil.; 3 Universidade de São Paulo, Faculdade de Medicina de Ribeirão Preto, Divisão de Pneumologia, Departamento de Clínica Médica, Ribeirão Preto, SP, Brasil.

**Dear Editor:**

Some patients with COVID-19 experience low oxygen levels without dyspnea. This paradoxical “happy hypoxemia” can worsen, and patients can recover within a short period; this has baffled both physicians and patients^1^. The pulse oximeter, a simple, noninvasive instrument used to estimate a patient’s peripheral arterial blood oxygen saturation (SpO_2_), has become a routine tool in the COVID-19 pandemic for emergency care units and domestic use. In April 2020, The New York Times published an article about the importance of having a tiny fingertip device at home to assess oxygen saturation, which complained about the scarcity of the device at that time^2^.

Due to the rise in the popularity of pulse oximeters, asymptomatic hypoxemia has become a common complaint of patients with leprosy under treatment during the pandemic. Dapsone, one of the antibiotics used in multidrug therapy (MDT/WHO) for leprosy, can cause acute hemolysis and methemoglobinemia (with or without symptoms) even at therapeutic doses, due to the continued oxidative stress caused by its metabolites^3^ ^,^ ^4^. This drug is also commonly used in dermatology to treat inflammatory and immunobullous diseases, such as pemphigus, pemphigoids, and neutrophilic dermatoses^3^.

This study primarily aimed to determine the adverse effects of dapsone on peripheral hypoxemia and its differential diagnosis with happy hypoxia encountered during the COVID-19 pandemic.

A descriptive observational case-series study was conducted involving 16 patients with leprosy who underwent multidrug therapy from a private clinic (PC). An individual fingertip oximeter was provided to each patient to randomly measure the SpO_2_ at home at least 12 times/day per month, noting each measure in a spreadsheet beyond the dapsone taking time. Furthermore, for a comparative analysis, a single SpO_2_ measure was documented 18 to 24 h after the daily intake of anti-leprosy medicines in 76 other patients followed up in the leprosy outpatient clinic at the Clinical Hospital (HC), during the monthly medical appointment, when they received the supervised MDT/WHO dose.

This study was approved by the Research Ethics Committee of the HC of Ribeirão Preto Medical School, University of São Paulo. Written informed consent was obtained from every participant, including the parent/guardian of those under 18 years of age. All procedures involving human subjects complied with the ethical standards of the Declaration of Helsinki (1975/2008).

**FIGURE 1:**
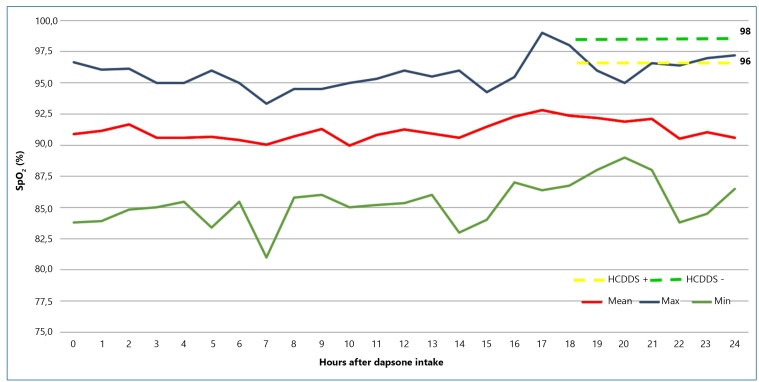
Distribution of SpO_2_ measurement averages taken at each hour after dapsone intake (mean, maximum, and minimum values) among leprosy patients.

Legend: Dashed lines represent the mean of a single SpO_2_ measurement after 18 to 24 h of intake of anti-leprosy medicines in Clinical Hospital patients using dapsone (HCDDS+, yellow) and not using dapsone (HCDDS−, green).

In 2020, 16 leprosy patients (8 males and 8 females, average age 55.8 years, range 6-80 years) were randomly investigated from PC using dapsone as part of the MDT/WHO. None of the patients had symptomatic anemia or a decrease in hemoglobin levels prior to dapsone use. The means of SpO_2_ measurements in each month of MDT/WHO were 86.6 (1^st^/n = 8), 90.9 (2^nd^/n = 6), 90.7 (3^rd^/n = 3), 93.2 (4^th^/n = 2), 94.1 (5^th^/n = 2), 94.6 (6^th^/n = 2), 91.6 (7^th^/n = 2), 92.4 (8^th^/n = 2), and 92.1 (9^th^/n = 2). The mean SpO_2_ was 91.2% (85.3%-95.8%) throughout the study period (Figure 1). All patients had an O_2_ saturation >95% on arterial blood gas tests. Only three patients presented with dyspnea, fatigue, and malaise, which improved when dapsone was replaced with ofloxacin.

Patients from HC who did not use dapsone (HCDDS−; n = 61) had a higher median SpO_2_ (98%) than those who received dapsone (HCDDS+; n = 15; 96%; p = 0.02; Figure 1). Both groups, HCDDS−/HCDDS+, presented higher SpO_2_ than that in the group that measured it regularly at home (p < 0.001).

The temporal relationship between the use and discontinuation of dapsone with the patient’s symptoms and level of SpO_2_ makes COVID-19 infection less likely in these patients as responsible for “happy hypoxia.” Among the patients from PC, 10 (62.5%) were tested for SARS-CoV-2 (COVID-19), and all were negative. Unfortunately, testing for COVID-19 infection was not performed in all patients, which is a limitation of our study.

These findings were detected only because the COVID-19 pandemic outbreak led to the fear of having “happy hypoxia,” in turn resulting in increased availability of pulse oximeters. During the COVID-19 pandemic, dermatologists should be aware of asymptomatic low SpO_2_ detected by fingertip devices in patients using dapsone, which may indicate the diagnosis of methemoglobinemia and/or sulfhemoglobinemia, the latter being a rare condition^5^.

Thus, caution should be observed as the effects of oxidative stress due to dapsone are not yet fully understood, and the risk of this fake sign of hypoxemia needs to be highlighted in these COVID-19 days.
